# Lutein and Zeaxanthin Intake during Pregnancy and Visual Function in Offspring at 11–12 Years of Age

**DOI:** 10.3390/nu14040872

**Published:** 2022-02-18

**Authors:** Martin J. Anderson, Dora Romaguera, Dave Saint-Amour, Serena Fossati, Silvia Fochs, Nuria Pey, Martine Vrijheid, Jordi Julvez

**Affiliations:** 1 School of Medicine, University of St Andrews, St Andrews KY16 9TF, UK; 2 Department of Ophthalmology, Princess Alexandra Eye Pavilion, NHS Lothian, Edinburgh EH3 9HA, UK; 3 ISGlobal, 08003 Barcelona, Spain; dora.romaguera@isglobal.org (D.R.); serena.fossati@isglobal.org (S.F.); silvia.fochs@isglobal.org (S.F.); nuria.pey@isglobal.org (N.P.); martine.vrijheid@isglobal.org (M.V.); jordi.julvez@iispv.cat (J.J.); 4 Health Research Institute of the Balearic Islands (IdISBa), 07120 Palma de Mallorca, Spain; 5 Consorcio Centro de Investigación Biomédica en Red-Fisiopatología de la Obesidad y Nutrición (CIBEROBN), Instituto de Salud Carlos III, 28029 Madrid, Spain; 6 Department of Psychology, Université du Québec à Montréal, Montreal, QC H2V 2S9, Canada; saint-amour.dave@uqam.ca; 7 Centro de Investigacion Biomedica en Red Epidemiologia y Salud Pública (CIBERESP), Instituto de Salud Carlos III (ISCIII), 28029 Madrid, Spain; 8 Department of Experimental and Health Sciences, Universitat Pompeu Fabra, 08003 Barcelona, Spain; 9 Institut d’Investigació Sanitària Pere Virgili (IISPV), Hospital Universitari Sant Joan de Reus, 43204 Reus, Spain

**Keywords:** visual acuity, contrast sensitivity, pregnancy, childhood, lutein, zeaxanthin, population-based birth cohort

## Abstract

(1) Background: Lutein and zeaxanthin (L&Z) are essential dietary nutrients that are a crucial component of the human macula, contributing to visual functioning. They easily cross the placental barrier, so that retinal deposition commences during foetal development. This study aims to assess associations between maternal L&Z intake during pregnancy and offspring visual function at 11–12 years. (2) Methods: Using the Spanish INfancia y Medio Ambiente project (INMA) Sabadell birth cohort, 431 mother–child pairs were analysed. L&Z data were obtained from food frequency questionnaires (FFQ) at week 12 and 32 of pregnancy, alongside other nutritional and sociodemographic covariates. Contrast vision (CS) and visual acuity (VA) were assessed using the automated Freiburg Acuity and Contrast Testing (FRACT) battery. Low CS and VA were defined as being below the 20th cohort centile. Associations were explored using multiple logistic regression. (3) Results: After controlling for potential confounders, L&Z intake during the 1st and 3rd trimester did not reveal any statistically significant association with either CS or VA in offspring at age 11/12 years. (4) Conclusions: No evidence of a long-term association between L&Z intake during pregnancy and visual function in offspring was found. Further larger long-term studies including blood L&Z levels are required to confirm this result.

## 1. Introduction

It is increasingly recognised that early life exposures, including maternal nutrition during pregnancy, play an important role in determining a child’s future health [1]. There appears to be a complex interaction of socio-economic and behavioural factors that mediate the effects of early life exposures [2,3]. Exploring nutritional exposures in utero that may impact long-term outcomes may therefore offer valuable targets in addressing sources of health inequality, given the link between nutrition and social determinants of health, such as socioeconomic status (SES), education and the underlying public health policy environment [4,5]. 

The plant derived pigments lutein and zeaxanthin (L&Z) are two dietary carotenoids, mainly found in vegetables, such as kale, leek and sweet peppers, and to some extent in eggs, fruits and other foods [6]. These carotenoids are not synthesized in humans and dietary intake is therefore the exclusive source [7]. Furthermore, these micronutrients are present in placental and umbilical cord blood as well as being actively transported into breastmilk [7,8]. Furthermore, placental L&Z appears strongly correlated with maternal plasma concentrations, and subsequently maternal dietary intake of these nutrients [9]. Examining maternal dietary intake during pregnancy may therefore allow an assessment of the impact of this in utero exposure to L&Z on the development of the offspring.

L&Z are uniquely concentrated at the human macula and fovea (the central portion of the retina responsible for high-resolution vision), forming part of the macular pigment. Retinal deposition of these carotenoids commences during foetal life [10,11] and may therefore be influenced by in-utero exposure to these nutrients as L&Z are transferred to the developing foetus via the placenta [7]. Foveal structures are recognisable as early as 11 weeks gestation with the foveal pit appearing at week 25, while histological maturity is usually reached in the second year of life [10,11,12,13]. This further supports the notion that L&Z intake during these time periods may impact macular and foveal development. 

L&Z are essential for adequate macula functioning and are thought to exert these effects through a number of mechanisms [10]. As yellow pigments, L&Z absorb high-energy short wavelength blue light hitting the retina and thus may prevent long-term photo-oxidative damage at the macula [14]. L&Z are believed to exert more general antioxidant effects at the retinal level, potentially mitigating other sources of oxidative stress, such as oxygen rich retinal blood vessels and broad spectrum visible light [12]. Filtration and absorption of the short-wavelength high energy blue light thus improve neural processing of visual inputs [14]. 

In adults, L&Z supplementation has been shown to improve visual performance, such as visual acuity (VA) and contrast sensitivity (CS), and diets high in L&Z appear protective against degenerative ocular diseases such as age-related macular degeneration (AMD) [15,16]. Although, the beneficial effect of L&Z supplementation seems to be attenuated when co-supplemented with poly-unsaturated fatty acids (PUFA) [17]. Interestingly, the incidence of AMD in later life appears higher in women who have had multiple pregnancies and L&Z levels were found to be significantly lower in multiparous women, indicating that depletion during pregnancy may contribute to this risk [18]. Thus, dietary intake of L&Z during pregnancy may be helpful in this group. 

A recent study by Lai et al. [19] is the first to describe an association between higher maternal L&Z blood levels at birth and lower likelihoods of poor VA in children at three years of age. Yet, there are currently no studies evaluating the longer-term effects of maternal L&Z intake during pregnancy and VA. Furthermore, other visual function such as CS might be more sensitive to detect more subtle functional improvements [20]. 

The aim of our study was therefore to evaluate whether maternal dietary intake of L&Z during pregnancy was associated with visual function (both VA and CS) at age 11–12 in their offspring, and whether factors such as SES, parity and L&Z intake of the offspring during childhood modify the association.

## 2. Materials and Methods

### 2.1. Study Population

The current research used data from the INMA cohort based in Sabadell, Catalonia, Spain. INMA is a multi-centre longitudinal birth cohort study that includes assessment of data from pregnancy to childhood. More detailed descriptions of the wider INMA project are provided elsewhere [21]. Study participants from the Sabadell birth cohort include women presenting to the public health centre of Sabadell for routine antenatal care services within the first trimester and their offspring, who were periodically followed up during childhood [21]. Participants were recruited over a two-year period between 2004 and 2006. Study inclusion criteria were maternal age > 16 years, singleton pregnancy, non-assisted reproduction, delivery scheduled in reference hospital and absence of communication barriers. The Sabadell cohort is the only INMA group with visual outcome data available. 

### 2.2. Exposure Variables—Lutein and Zeaxanthin Estimation

Semi-quantitative Food Frequency Questionnaires (FFQ) of 101 food items were used to assess usual dietary intake of participating pregnant women at recruitment during the 1st trimester and at a further 3rd trimester visit. The FFQ is based on the ‘Harvard Questionnaire’ [22] and has been adapted and validated for a Spanish context in the Valencia INMA cohort [23]. Participants were prompted to report intake of specific foods using reference portions and nine frequency categories ranging from never to more than six times per day. For the first visit, women were asked to report their usual intake since the last menstrual period. During the second visit they were asked to report on intake between the first and third trimester. During a later follow up visit, a further FFQ estimated L&Z intake of the offspring at age 4. The reported food intakes were then used to estimate the average daily intake of L&Z in milligrams (mg). As current nutrient databases report L&Z together, they were assessed as one combined exposure. Nutrient information from the FFQ was derived using the United States Department of Agriculture food-composition tables [24], and further information on specific foods was obtained from a Spanish food table [25]. Total energy-adjusted intake estimates (using the residual method) were used to reduce confounding and extraneous variation [26].

### 2.3. Outcome Variables—Visual Acuity and Contrast Sensitivity

CS and VA were measured in study participants at age 11–12 years using the automated Freiburg acuity and contrast test (FRACT), a validated computerised visual screening battery [27]. Children were sat at a distance of two metres from a calibrated computer screen and indicate optotype positions on a keyboard. Ambient lighting conditions in the testing room were measured (in Lux) using a handheld light meter. Any issues regarding testing were recorded as free text. Binocular contrast thresholds were established by displaying optotypes (known as Landolt Cs or rings) in the center of a computer screen at various contrast levels. Participants were asked to indicate the position of the optotype gap (left, right, up or bottom) via a keyboard. The least amount of contrast required to detect the optotype gap specifies the contrast threshold, with lower values indicating better contrast vision. CS was obtained as the reciprocal of the contrast threshold and converted to a logarithmic unit, the logarithm of Weber contrast sensitivity (logCS_Weber_) [28]. Higher values of logCS_Weber_ indicate better contrast vision. Refractive correction (glasses) were used by participants for contrast testing, if a history of refractive error was present. VA was assessed using the same FRACT test. In this case, optotypes of varying sizes, but constant contrast (100%), were displayed. The size of the gap at the best level of vision (smallest optotype), measured in minutes of arc, provides the minimum angle of resolution (MAR) and the logarithm of this value forms the standard acuity measure of logMAR [27,28]. Lower values indicate better vision. Refractive correction was not worn during VA assessment. 

### 2.4. Covariates

Potential confounders were defined a priori based on previous literature and are outlined below. Sex, birthweight (in grams (g)) and gestation (in weeks) were recorded by midwives at the time of delivery, as was maternal age (in years). Low birthweight and prematurity can be a result of intrauterine insults and other congenital co-morbidities, which can affect visual function and therefore warrant consideration [29]. Increasing maternal age is associated with higher risks of pregnancy itself, as well as higher rates of refractive error in offspring [30]. As multiparity has been associated with potential L&Z depletion, the degree of parity documented at recruitment was also analysed [18]. Maternal alcohol consumption and smoking status during pregnancy were recorded at the time of the FFQs in trimester 1 and 3 and coded as any smoking during pregnancy (yes/no) or any alcohol consumption during pregnancy (yes/no). Exposure to either substance in utero has previously been linked to deleterious visual outcomes in children [31]. 

Parental SES and education have been linked to variation in visual outcomes in children [29,32]. Data on SES in our cohort is described in the form social class defined by occupation and maternal education (primary/secondary/university). Occupation-based social class was adapted from the international ISCO88 classification of occupations and divided into high (status I–II includes managers and technicians), medium (status III includes skilled) and low (IV–V semiskilled/unskilled) based on maternal occupation at recruitment [33]. Although breastfeeding status has not conclusively been shown to affect long-term visual function [34,35], L&Z are present in breastmilk, thus contributing to L&Z exposure of newborns and infants, and thus potentially impacting visual development [8]. Breastfeeding was assessed during interviews at 6 and 14 months postnatally and analysed as a continuous variable (weeks) [36]. Furthermore, maternal intake during pregnancy of other nutrients, such as PUFA (mg/day), which may impact visual development [37], as well as bioavailability of L&Z [17] was also obtained from the FFQs. 

Parental and childhood history of eye disease is strongly related to visual functioning [29]. Data on parental and child history of eye disease were collected via screening questionnaires administered at the same visit as visual testing (child aged 11–12 years). A history of childhood eye disease was deemed present if a history of any of the following was recorded: ametropia (a refractive error), colour blindness or amblyopia (reduced VA caused by abnormal visual development).

### 2.5. Statistical Analysis and Model Selection

As dietary estimations are unlikely to precisely represent the true intake, categories in the form of tertiles of L&Z consumption were created. Here, those categorised in high and low tertiles are more likely to represent truly high and low consumptions. 

Sociodemographic variables were compared between the study cohort and those lost to follow up. For continuous variables a student’s *t*-test was used to compare two groups, and ANOVA for tertile group comparisons. A chi-squared test (CHI^2^) test was used to evaluate differences for categorical variables, and the Fisher exact test was used if the expected frequency of a variable in a group was less than five. 

The outcome variables were categorised into binary outcomes (low visual acuity—yes/no; low contrast sensitivity—yes/no) using the lowest 20th centile within each outcome as the cut off. For VA, the cut off was −0.02 logMAR and 1.77 logCS_Weber_ for CS. Logistic regression was then performed to identify the adjusted odds ratio (OR) of low CS and VA per L&Z tertile at the first and third trimester with three different models. Test for trend was then performed using the mean values of consumption of each L&Z tertile within a given regression model. ORs are given alongside 95% confidence intervals. Three multiple logistic regression models were used. A minimally adjusted model (Model A) adjusted for age at eye testing and sex only. Model B additionally included maternal age, gestational age at birth, birthweight, maternal smoking during pregnancy, alcohol during pregnancy, PUFA intake, social class, breastfeeding duration and total energy intake. Model B covariates were selected as they were deemed necessary to control for confounding based on a directed acyclic graph (DAG) (see Supplementary Figure S1) that was constructed using the online DAGitty tool [38]. Model C further adjusted for history of childhood eye disease, lighting conditions at eye test and childhood L&Z intake at age 4, as these were felt to warrant further analysis given their potential impact on any observed association. 

As multiparity may be associated with depleted maternal L&Z stores [18], parity was assessed as a potential effect modifier, to identify if multiparous mothers benefit more from higher dietary intakes. Additionally, effect modification was assessed for breastfeeding duration. SES influences childhood exposures and outcomes in multifactorial and complex ways [2]. We therefore also tested for interaction of social class (manual vs. non-manual occupation) and L&Z intake to assess whether L&Z intakes impacted visual function differently based on the socio-economic context of the child. Interaction was also assessed for offspring sex. *p*-values for interaction were computed for each scenario rerunning Model C with a multiplicative interaction term inserted between the L&Z intake variable and the variable of interest, using the Wald test to assess for significance of interaction.

A significance level of 0.05 was deemed statistically significant. Statistical analyses were performed using Stata software (StataCorp. 2019. Stata Statistical Software: Release 16. College Station, Texas: StataCorp LLC.).

### 2.6. Ethical Clearance

Ethical approval for data collection and analysis for the INMA project was obtained from the Hospital of Sabadell and the Municipal Institute of Medical Research (No 2005/2106/I). Parental written informed consent was obtained for all participants.

## 3. Results

### 3.1. Cohort Profile

A total of 657 women were recruited during pregnancy. Of those, 42 had missing nutritional data on L&Z intake during pregnancy. During the follow up period, a further 177 participants (offspring) were lost to follow-up (LTF) prior to visual outcome testing at age 11/12 years. The reasons were not specified. Five children could not complete the FRACT testing procedure due to unspecified technical or behavioural issues, and two participants had visual outcome values outwith physiologically plausible limits and were thus also excluded. Thus, 431 mother–child pairs were included in the analysis. 

Mean maternal age at first visit was 31.9 years (SD 4.1), with the vast majority being of white ethnicity (98.4%). Overall, 66.3% (*n* = 283) reported completing secondary education or less, and 42.4% (*n* = 182) were of low social class (*n* = 182, 42.4%). Offspring were 51.5% (*n* = 222) male and 48.5% female (*n* = 209).

As one must assume that the reason for LTF were not random, covariates between those included and those excluded (LTF and those with technical testing issues) in the analysis are compared in Supplementary Table S1. Briefly, the study cohort was significantly older (*p* < 0.001) with an average age of 31.9 years (SD 4.1) compared to those excluded, average age 30.5 (SD 4.8). There were significant differences in education (*p* < 0.001) and social class (*p* < 0.001) with those LTF tending to be of lower social class and with lower levels of education. In addition, there was a higher proportion of those with a non-white ethnic background (6.7% vs. 1.6%, *p* < 0.001) in the LTF group. L&Z intake was not significantly different between groups at week 12 (*p* = 0.8591) or 32 (*p* = 0.6060). The birthweight of offspring in the study cohort was significantly higher (*p* = 0.0098) at 3271 g (SD 405) compared to those excluded (3159 g, SD 518) and breastfeeding duration was longer (*p* = 0.0192), by around two weeks on average. 

Table 1 outlines the distribution of covariates amongst the study cohort by L&Z intake tertile groups at week 12 (1st trimester). Significant differences between L&Z intake tertile groups were found for total energy intake (*p* = 0.0129), intake being lower amongst the lowest tertile, and maternal age (*p* = 0.0097), where the lowest intake tertile group was younger on average. 

Findings at week 32 (3rd trimester) indicated that those in higher intake tertiles were slightly older (*p* < 0.0001) and tended to be multiparous (*p* = 0.029). Furthermore, there were significant differences in education (*p* = 0.016) and social class (*p* = 0.022), with the lowest intake tertile having the highest proportion of mothers in the low social class and lowest level of education. The distribution of covariates at week 32 is further elaborated in Supplementary Table S2. Supplementary Table S3 further outlines the distribution of study covariates based on visual outcome category. 

### 3.2. Lutein and Zeaxanthin Intake during Pregnancy and Visual Function

Table 2 outlines the ORs (95% CI) of low CS and VA associated with inakes of L&Z, within the three specified multiple logistic regression models. No statistically significant trend for L&Z intake tertiles and visual outcomes was observed at week 12 or 32. Point estimates of ORs for higher L&Z intake tertiles (tertiles 2 and 3) often tended to be above 1, especially at week 12, suggesting a potentially deleterious effect on visual function, although the confidence intervals are very wide, coupled with *p* values well above 0.05. Furthermore, estimates of association tended to not change substantially between models, apart from VA in Model C, in which most ORs point estimates non-significantly dipped below one at week 12 and 32 likely due to the additional adjustment for eye disease, as this will have taken into account the fact that children, unlike during CS testing, did not wear refractive correction during VA testing. 

The ORs of low VA and CS associated with childhood L&Z intake at age four are presented in Table 3. Results did not show a significant trend for intake tertiles for associations with CS (week 12, *p* = 0.2167; week 32, *p* = 0.2019) and VA (week 12, *p* = 0.6640; week 32, *p* = 0.6367). Overall, point estimates for CS indicated a non-significant protective effect against low CS with ORs below 1 while for VA point estimates were above 1 for the second and third intake tertiles in reference to the lowest intake tertile. 

### 3.3. Effect Modification

No statistically significant interactions between L&Z intake at week 12 or 32 and social class, breastfeeding or offspring sex, were identified, and further stratified analysis was therefore not conducted. Supplementary Table S4 gives further details for the different variables assessed for interaction at both time points. Parity appeared to significantly interact with L&Z intake at both CS (0.0052) and VA (0.0254) at week 32 but not at week 12 (CS, *p* = 0.2759; VA, *p* = 0.3267). Stratified analysis for week 32 by parity level is shown in Table 4.

Higher intake tertiles of L&Z tended to be a protective factor against low VA and CS in multiparous women, while for nulliparous women, the highest intake tertile appeared to be associated with a significantly increased odds of low VA and CS, in contrast to the hypothesis that higher intakes are a protective factor.

## 4. Discussion

### 4.1. Summary

To our knowledge, this is the first study to examine the long-term association between maternal L&Z intake during pregnancy, as well as childhood L&Z intake, and visual function in school-aged offspring. Overall, maternal intake of L&Z assessed during the first and third trimester was not significantly associated with either CS or VA in our cohort. There was no evidence of a crude association between L&Z intake during pregnancy and either visual outcome; this did not change when adjusting for maternal, child and visual covariates during logistic regression modelling. Similarly, higher childhood L&Z intake at age four also did not show any significant association with visual outcomes.

For both CS and VA, interaction between L&Z intake and social class, breastfeeding and sex was not statistically significant at week 12 or 32, while parity showed a significant interaction at week 32 only. Counterintuitively, stratified analysis showed significantly higher risks of low VA and CS for nulliparous women in higher L&Z intake tertiles, while for multiparous women higher L&Z intakes tended to be protective.

### 4.2. Context

The time periods during pregnancy assessed in this study correspond to periods of significant development of the human macula and fovea in utero [11,13]. These structures are crucial for development of good VA and CS [14].

Lutein supplementation in term infants has been shown to have a beneficial effect on levels of oxidative stress [39]. The developing retina in the newborn is particularly susceptible to damaging effects of excessive light and associated oxidative stress, which supports the hypothesis of a protective effect of higher levels of the antioxidants L&Z, as these are uniquely concentrated in the retina [40]. As retinal deposition of L&Z begins early in utero and L&Z readily crosses the placenta, maternal L&Z intake is hypothesized to affect L&Z-related retinal development [7,12,14]. 

Further research has highlighted a correlation between plasma lutein levels and full field electroretinogram response amplitude in rod photoreceptors, as well as rod photoreceptor sensitivity after four months of lutein supplementation in preterm (< 33 weeks) infants [41]. Increased oxidative stress seen in preterm infants is thought to explain the benefits of lutein, an antioxidant, seen in this group [40]. Additionally, L&Z are thought to reduce abnormal vascular endothelial growth factor (VEGF) expression, a key driver of retinal degenerative diseases such as AMD [42], but also diseases such as retinopathy of prematurity [43].

Interestingly, a recent meta-analysis did not show any effect on retinopathy of prematurity, sepsis or mortality of lutein and zeaxanthin supplementation in preterm (< 32 weeks) infants [43]. 

In healthy adults, L&Z intake appears to correlate with macular pigment optical density (MPOD) [9,17,44]. MPOD in turn has been shown to provide an indication of the amount of L&Z at the macula [40]. In healthy young adults, L&Z supplementation has been shown to improve MPOD and benefit contrast sensitivity, visual processing speed, and visual fatigue when compared to placebo [45,46,47]. 

Our study findings contrast with results recently published by Lai et al., which examined 471 mother–child pairs in Singapore to assess the association between maternal lutein and zeaxanthin blood concentrations at delivery and uncorrected VA of offspring at age three [19]. Results indicated a statistically significant reduction in likelihood of low VA (logMAR > 0.3) for the highest tertile of zeaxanthin, as well as the middle tertile for lutein concentration at birth. 

There are a number of potential factors that may have contributed to the differing findings. Overall, 126/471 children in their cohort had poor VA, which is high and likely related to uncorrected refractive error, the commonest cause of visual impairment in children, especially in the region studied [32]. No clear physiological relevance of L&Z in the pathogenesis of refractive error has been described in contrast to macular function [10,14]. As in our study, the fact that uncorrected VA was used as an outcome means that one cannot ascertain the relative contributions of refractive error and macular function, the latter being more relevant to L&Z physiology. Our CS measurement was performed with refractive correction and may therefore be seen as a more relevant marker of macular function than uncorrected VA. 

In our study, the food composition database did not differentiate between L&Z as these are usually found in similar foods and usually classified together so we are unable to examine the individual effect of each nutrient separately, as was done in the study by Lai et al. [19]. A further major difference was that rather than using intake estimates, in the study by Lai et al., blood concentrations were used, which are inherently more accurate. However, while biomarkers would have given a more accurate point estimates of L&Z levels of the mother, blood levels alone may not have provided information on clinically relevant nutritional intake requirements to obtain an effect. Using dietary intake is not only non-invasive, but also provides the opportunity to estimate clinically relevant dietary factors within the complex interaction of a person’s diet as a whole. Future approaches should attempt to combine FFQs and blood markers to gain a more complete picture.

The absence of a significant association between L&Z intake and visual function in our cohort may also be due to the substantially longer follow up duration, in which additional factors may be more relevant drivers of ultimate visual outcomes rather than maternal L&Z intake. Putative beneficial effects of L&Z on vision in offspring might manifest only in early life and disappear as the visual system matures at age 11–12, providing useful information on the relative long-term importance of L&Z intake during pregnancy. This possibility has been observed with milk-based DHA supplementation in premature infants, where improvement in VA was observed in infancy but not thereafter [48]. 

In addition, the critical period for childhood visual development appears to be around 7–8 years of age, although evidence suggests it may even be slightly longer [49]. The follow up at age 11–12 years is therefore a useful timepoint as it includes the most important critical period of a child’s visual development. 

To further examine the effect of childhood L&Z intake as a potentially important marker during the critical period for future visual function, we also analysed childhood L&Z intake at age four. Even though, this may not be an accurate representation of childhood nutrition throughout the first 12 years, it provides a meaningful point estimate in time and revealed no association between childhood L&Z intake at age four years and visual outcomes.

Ultimately, our findings are in keeping with no or a small non-clinically significant association between maternal L&Z and child visual function. Overall, despite Lai et al.’s recent findings, there is little long-term evidence of the impact of maternal nutrition during pregnancy on offspring functional visual outcomes beyond physiological plausibility and effects on biological markers rather than function [9,14,19,31,37,50]. There may be a publication bias against null findings and assessing specific dietary factors within the complex interaction of exposures in utero and in childhood are likely to bias research findings toward the null hypothesis. 

However, other studies in adults suggest that higher L&Z intake later in life not only increases macular pigment density but is also associated with clinically relevant improvements in CS, as well as protection against AMD [14,15,17,51]. In combination with evidence of high L&Z concentration in the developing retina [10], one would expect higher L&Z intake to be relevant in visual function throughout the lifespan. However, many of the studies in adults rely on high dose supplementation, which is not comparable to intake levels seen in our cohort. For instance, the AREDS2 formulation is a well-studied and widely used supplement to prevent progression of AMD and contains 10mg and 2mg of lutein and zeaxanthin, respectively, as a daily dose [14]. This is substantially higher than the mean of our highest combined L&Z intake tertile at week 12 (3.5 mg/day) and 32 (3.4 mg/day) and raises the question whether much higher doses of L&Z are required to consistently observe clinically meaningful differences. 

Further, the Spanish median daily L&Z intake of 3.25 mg/day, which is comparable to intake in our cohort, is substantially higher than that in other countries such as the United Kingdom (median 1.19 mg/day) [52] and the United States (1–2 mg/day) [53]. There may be a minimum L&Z requirement for visual development, beyond which only minimal benefit is seen unless very high doses are supplemented. The physiological effects and relative importance of L&Z intake are likely to differ across the life span, as antioxidant stores including lutein and zeaxanthin accumulate and subsequently deplete in adult life, increasing the risk of macular disfunction and degeneration [54].

Nutrition is heavily influenced by social determinants of health [2,4,5] and may therefore contribute to and perpetuate resulting health inequity. The significant differences between the study and LTF cohort in social class, education and ethnicity likely highlight some health determinants relevant in the population studied. Interestingly, in our cohort at week 12, there was no difference in social class or education by L&Z tertile. However, by week 32, there was a significant difference in both maternal education and social class amongst L&Z tertiles with those more educated and in higher social classes more likely to be in the higher intake tertile. Given that L&Z are predominantly found in foods considered important for a healthy and balanced diet [52], there is the potential that diet-related health literacy on the importance of a healthy diet during pregnancy is greater in those in higher socio-economic categories and efforts need to be made to ensure all pregnant women receive suitable and tailored support on nutrition during pregnancy to ameliorate some of the effects of lower socio-economic status on pregnancy-related health [4]. 

In further analysis, social class was not found to be a significant effect modifier of either visual outcome. Breastfeeding, a crucial source of L&Z in infancy [55], was also found not to be a significant effect modifier. Maternal diet during breastfeeding was not assessed and may influence the L&Z exposure in breastmilk [8,55]. However, one can assume that diet during pregnancy is strongly correlated with diet post-partum, making breastfeeding a relevant marker of L&Z exposure when taking intake during pregnancy into account.

Parity, on the other hand, was found to significantly interact with L&Z intake, and further stratified analysis revealed counterintuitive results as higher L&Z intakes were associated with significantly increased odds of poor VA and CS in nulliparous women contrary to the proposed beneficial effects of L&Z on retinal development and on-going health [10,14,15,19]. Higher L&Z intakes were non-significantly associated with a protective effect in multiparous women. Given the evidence that multiparity can cause L&Z depletion in mothers [18], these findings support the suggestion that those depleted in L&Z may benefit most from higher L&Z intakes. Nevertheless, this would not explain the counterintuitively high ORs in nulliparous women. This may well represent a type II error given the overall number of statistical tests performed and the relatively small number of women within each parity category. 

### 4.3. Strengths and Limitations

#### 4.3.1. Study Design

A major strength of this study is its long-term prospective design, alongside the availability of numerous detailed covariates on socio-demographic and nutritional factors, which could be adjusted for, long-term follow-up, and a relatively low LTF rate (29%) given the length of the study. The socio-demographic differences between the study cohort and those LTF indicate the latter were significantly younger, of lower social class, breastfed for a shorter period and had offspring with slightly lower birthweight on average. Further, the proportion of women of a non-white background was significantly higher in the LTF group, although the absolute number of non-white participants in both groups was small. Overall, this suggests there is the potential of selection bias as the ultimate study cohort may not have been truly representative of the population studies. Furthermore, the single centre, predominantly white, cohort limits generalisability of findings to other contexts and the sample size was relatively small. Therefore, estimates of association were broad, reflected in very wide confidence intervals, and subtle associations may have gone undetected. There are a number of additional factors that may have influenced the study findings and are outlined in further detail below.

#### 4.3.2. Exposure Estimation

Previously, average L&Z intake in Spain has been estimated at 3.25 mg/day [52], which is similar to the mean intake at week 12 (3.52 mg/day) and week 32 (3.44 mg/day) in our study cohort. The average Spanish intake would have fallen within the second tertiles at both week 12 and 32, as expected. This suggests that our 1st and 3rd tertiles are likely representative of lower and higher than national average L&Z consumptions, respectively. Further, pregnant women are likely to be more aware of their diet during pregnancy as some foods and supplements are not suitable during this time. One would expect this to improve the recall in food frequency questionnaires.

However, there are a number of limitations of estimating nutritional intakes using FFQs. Food intake estimation is subject to recall bias and may therefore over- or underestimate actual intake. Furthermore, absorption of L&Z may be affected by other dietary and physiological factors, and dietary intake thus may not correlate with blood concentrations in a similar fashion across individuals [16]. For instance, adding PUFA to L&Z supplementation has been shown to be less effective at preventing macular degeneration than L&Z alone [17], and PUFA intake was therefore included in our modelling. Other factors, such as the type of L&Z containing food, and whether they are cooked, processed or eaten concomitantly with other fatty acids are also thought to influence bioavailability [12], but this level of detail is hard to ascertain and utilise effectively using the FFQ alone. Blood levels of carotenoids would have provided further detail but were not available for this study. However, a recent study investigating the association between maternal L&Z intake, assessed using a FFQ, and cognitive function in offspring found a significant association, despite not adjusting for any co-variates affecting L&Z bioavailability, indicating that L&Z intake alone may still be a reasonable indicator of L&Z’s effects on the developing child [56]. 

Additionally, the FFQ used in this study has been extensively validated elsewhere, as well as within the INMA cohort of Valencia [22,23]. As intakes of many nutrients are correlated with total energy consumption, there may be a non-causal association which is confounded by total energy intake. To address this, we adjusted for total energy intake which is likely to reduce confounding and reduce the impact of extraneous variation of a nutrient due largely to variation in total energy intake [26]. However, the inherent imprecision of nutrient estimation is likely to have biased our findings towards the null hypothesis (no association between L&Z and visual outcomes) [26]. 

#### 4.3.3. Visual Function Assessment

The FRACT testing battery has been extensively validated previously when a standardised set up was used [27]. For the measurements included in the analysis, no testing issues were reported, but other extraneous circumstances causing measurement bias, such as unrecorded differences in FRACT testing set ups, causing residual confounding cannot be assessed or excluded. The use of uncorrected VA is a likely source of bias. In addition, the physiological relevance L&Z in the pathogenesis of refractive error is unclear, while its role in macular development and function is more established [10,14]. 

Contrast sensitivity, as a more precise marker of macular function, was therefore also used. Contrast sensitivity testing was performed with refractive correction, making it a more reliable indicator of visual function as uncorrected refractive error as a source of bias is significantly reduced. 

Physiological markers, such as macular pigment density, which may have further quantified the relationship between L&Z intake and macular status, were not available for the study cohort. Nevertheless, VA and CS provide a clinically relevant and robust functional assessment of vision.

Furthermore, visual function is influenced by both ocular and cerebral function. Cerebral visual impairment is the most common cause of irreversible visual impairment in children and is associated with prematurity and other pre- and perinatal insults [29]. Even though history of ocular disease and prematurity was assessed, subtle undiagnosed cerebral impairment may be missed and therefore confound the results.

## 5. Conclusions

Our INMA birth cohort study based in Sabadell, Spain, did not find a significant association between maternal L&Z intake during pregnancy and visual outcomes in the form of CS and VA, in their offspring at age 11–12 years. The L&Z intake estimates were based on previously validated FFQ-based methods [23] and results were in keeping with previous estimates of average Spanish L&Z consumption [52]. However, the sample size was relatively small and may not have been able to detect more subtle effects on vision in our cohort. 

Given the increasingly recognized importance of nutrition, including L&Z intake, on offspring health [56] and longer-term health inequities [5], further larger long-term studies examining maternal nutritional factors, such as L&Z intake, which include blood concentrations for more precise exploration, are warranted. This will allow evidence-based health promotion and policy decisions to address and mitigate early life nutritional sources of health inequities. 

## Figures and Tables

**Table 1:** Baseline characteristics of participants according to tertile of lutein and zeaxanthin (L&Z) consumption during the first trimester (12-week assessment) of pregnancy. Based on the Sabadell cohort of the Spanish Childhood and Environment (INMA) project.

KERRYPNX	*n*	Total	1st Tertile (Low)	2nd Tertile (Medium)	3rd Tertile (High)	*p* Value
Maternal Covariates						
Lutein and Zeaxanthin Intake (mg/day)	429		143	143	143	
Mean (SD)		3.5 (2.0)	1.7 (0.5)	3.1 (0.3)	5.7 (1.8)	
Rank		0.6–11.5	0.6–2.5	2.5–3.7	3.7–11.5	
Total Energy Intake (kcal/day)	429					
Mean (SD)		2050.3 (482.0)	1997.9 (510.0)	2146.9 (493.6)	2006.0 (427.1)	0.0129 ^a^
Age (years)	429					
Mean (SD)		31.9 (4.1)	31.1 (4.3)	32.3 (4.0)	32.4 (3.9)	0.0097 ^a^
Parity, *n* (%)	427					
Nulliparous		250 (58.6)	89 (62.7)	82 (57.8)	79 (55.2)	0.432 ^b^
Parity 1 or more		177 (41.5)	53 (37.3)	60 (42.3)	64 (44.8)	
Ethnicity, *n* (%)	429					
White		422 (98.4)	140 (97.9)	141 (98.6)	141 (98.6)	1.00 ^c^
Other Ethnic Group		7 (1.6)	3 (2.1)	2 (1.4)	2 (1.4)	
Maternal Education, *n* (%)	427					
Primary or less		102 (23.9)	37 (25.9)	28 (19.7)	37 (26.1)	0.718 ^b^
Secondary		181 (42.4)	58 (40.6)	64 (45.1)	59 (41.6)	
Tertiary		144 (33.7)	48 (33.6)	50 (35.2)	46 (32.4)	
Social Class, *n* (%)	429					
High		104 (24.2)	37 (25.9)	29 (20.3)	38 (26.6)	0.544 ^b^
Medium		143 (33.3)	42 (29.4)	52 (36.4)	49 (34.3)	
Low		182 (42.4)	64 (44.8)	62 (43.4)	56 (39.2)	
Smoking during pregnancy, *n* (%)	429					
No		314 (73.2)	104 (72.7)	98 (68.5)	112 (78.3)	0.172 ^b^
Yes		115 (26.8)	39 (27.3)	45 (31.5)	31 (21.7)	
Alcohol during pregnancy, *n* (%)	429					
No		331 (77.2)	115 (80.4)	103 (72.0)	113 (79.0)	0.194 ^b^
Yes		98 (22.8)	28 (19.6)	40 (28.0)	30 (21.0)	
Maternal PUFA Intake (g/day)	422					
Mean (SD)		14.2 (3.1)	14.4 (3.1)	14.1 (2.9)	14.1 (3.3)	0.5520 ^a^
Child Covariates						
Sex, n (%)	429					
Female		209 (48.7)	69 (48.3)	77 (53.9)	63 (44.1)	0.251 ^b^
Male		220 (51.3)	74 (51.8)	66 (46.2)	80 (55.9)	
Prematurity (< 37 weeks gestation)	429					
No		419 (97.7)	139 (97.2)	142 (99.3)	138 (96.5)	0.362 ^c^
Yes		10 (2.3)	4 (2.8)	1 (0.7)	5 (3.5)	
Gestation (weeks) at birth	429					
Mean (SD)		39.7 (1.4)	39.8 (1.4)	39.8 (1.3)	39.6 (1.4)	0.1994 ^a^
Birthweight	429					
Mean (SD)		3269.6 (405.6)	3287.8 (425.8)	3266.0 (396.0)	3254.9 (396.6)	0.7851 ^a^
Predominant Breastfeeding (weeks)	428					
Mean (SD)		12.8 (9.5)	12.3 (9.9)	13.4 (9.1)	12.8 (9.5)	0.6421 ^a^
Lutein and Zeaxanthin Intake Age 4 (mg/day)	375					
Mean (SD)		0.9 (0.5)	0.8 (0.4)	1.0 (0.42)	1.0 (0.5)	< 0.00001 ^a^
Vision Covariates						
Parental History of Eye Disease, *n* (%)	420					
None		86 (20.5)	26 (18.4)	30 (21.3)	30 (21.7)	0.179 ^b^
One Parent		179 (42.6)	53 (37.6)	59 (41.8)	67 (48.6)	
Both parents		155 (36.9)	62 (44.0)	52 (36.9)	41 (29.7)	
Childhood History of Eye Diseased, *n* (%)	421					
No		330 (78.4)	115 (81.0)	106 (75.2)	109 (79.0)	0.484 ^b^
Yes		91 (21.6)	27 (19.0)	35 (24.8)	29 (21.0)	
Age at Eye Test (years)	429					
Mean (SD)		11.2 (0.5)	11.1 (0.50)	11.1 (0.54)	11.3 (0.5)	0.0288 ^a^

^a^—*p*-value from ANOVA ^b^—*p*-value from chi-squared test ^c^—*p*-value from Fisher exact test.

**Table 2:** Multiple adjusted analysis of the association between maternal L&Z intake during pregnancy and low contrast sensitivity and visual acuity based on the lowest 20th centile cut off in the Sabadell cohort of the Spanish Childhood and Environment (INMA) project.

	**Contrast Sensitivity**		
	Model A ^a^	Model B ^b^	Model C ^c^
Week 12	OR (95%CI)	OR (95%CI)	OR (95%CI)
1st Tertile L&Z	Ref	Ref	Ref
2nd Tertile L&Z	1.22 (0.69, 2.28)	1.21 (0.66, 2.23)	1.45 (0.74, 2.85)
3rd Tertile L&Z	1.31 (0.73, 2.32)	1.38 (0.76, 2.52)	1.63 (0.83, 3.22)
*p*-Trend	0.6432	0.5757	0.3450
Week 32			
1st Tertile L&Z	Ref	Ref	Ref
2nd Tertile L&Z	0.78 (0.43, 1.39)	0.75 (0.40, 1.39)	0.73 (0.38, 1.41)
3rd Tertile L&Z	1.17 (0.67, 2.05)	1.25 (0.70, 2.23)	1.38 (0.72, 2.62)
*p*-Trend	0.3742	0.2466	0.1700
	**Visual Acuity**		
	Model A ^a^	Model B ^b^	Model C ^c^
Week 12	OR (95%CI)	OR (95%CI)	OR (95%CI)
1st Tercile L&Z	Ref	Ref	Ref
2nd Tercile L&Z	1.27 (0.71, 2.30)	1.22 (0.66, 2.26)	0.84 (0.38, 1.85)
3rd Tercile L&Z	1.51 (0.84, 2.73)	1.38 (0.75, 2.53)	1.02 (0.45, 2.23)
*p*-Trend	0.3827	0.5861	0.8731
Week 32			
1st Tercile L&Z	Ref	Ref	Ref
2nd Tercile L&Z	1.26 (0.71, 2.25)	1.11 (0.59, 2.10)	0.67 (0.31, 1.48)
3rd Tercile L&Z	1.23 (0.68, 2.22)	1.27 (0.68, 2.35)	0.82 (0.36, 1.85)
*p*-Trend	0.6949	0.7500	0.6136

^a^—Model A: Logistic regression model adjusted for child age and sex. ^b^—Model B: Logistic regression model additionally adjusted for alcohol during pregnancy, smoking during pregnancy, gestational age, birthweight, maternal age, total energy intake, breastfeeding duration, socio-economic status, total maternal polyunsaturated fatty acids intake, parity. ^c^—Model C: Logistic regression model additionally adjusted for history of childhood eye disease, luminosity at eye test sight, childhood lutein and zeaxanthin intake at age four. Ref—referent category; OR—odds ratio.

**Table 3:** Multiple adjusted analysis of the association between childhood lutein and zeaxanthin intake at age four years and low contrast sensitivity and visual acuity based on the lowest 20th centile cut off in the Sabadell cohort of the Spanish Childhood and Environment (INMA) project.

	Contrast Sensitivity	Visual Acuity
	Model C ^a^	Model C ^a^
	OR (95%CI)	OR (95%CI)
1st Tertile L&Z age 4	Ref	Ref
2nd Tertile L&Z age 4	0.60 (0.31, 1.14)	1.43 (0.66, 3.10)
3rd Tertile L&Z age 4	0.62 (0.32, 1.20)	1.26 (0.56, 2.84)
*p*-Trend	0.2167	0.6640

^a^—Multiple adjusted logistic regression using Model C: child age, sex, alcohol during pregnancy, smoking during pregnancy, gestational age, birthweight, maternal age, total energy intake, breastfeeding duration, maternal lutein and zeaxanthin intake during pregnancy at week 12, socio-economic status, total polyunsaturated fatty acids intake, history of childhood eye disease, luminosity at eye test sight; Ref—referent category; OR—odds ratio.

**Table 4:** Stratified multiple adjusted analysis by parity (nulliparous vs. multiparous) of the association between maternal lutein and zeaxanthin intake and low contrast sensitivity and visual acuity based on the lowest 20th centile cut off in the Sabadell cohort of the Spanish Childhood and Environment (INMA) project.

Low Contrast Sensitivity		Low Visual Acuity	
	Model C ^a^		Model C ^a^
Week 32		Week 32	
Parity 0 (*n* = 200)		Parity 0 (*n* = 201)	
1st Tercile L&Z	Ref	1st Tercile L&Z	Ref
2nd Tercile L&Z	0.95 (0.34, 2.63)	2nd Tercile L&Z	1.21 (0.38, 3.90)
3rd Tercile L&Z	3.44 (1.39, 8.51)	3rd Tercile L&Z	1.95 (0.61, 6.19)
*p*-Trend	0.0079	P-Trend	0.5099
Parity 1+ (*n* = 140)		Parity 1+ (*n* = 141)	
1st Tercile L&Z	Ref	1st Tercile L&Z	Ref
2nd Tercile L&Z	0.43 (0.16, 1.17)	2nd Tercile L&Z	0.20 (0.05, 0.74)
3rd Tercile L&Z	0.39 (0.14, 1.14)	3rd Tercile L&Z	0.21 (0.05, 0.85)
*p*-Trend	0.1526	P-Trend	0.0351

^a^—Model C: Logistic regression model adjusted for child age and sex, alcohol during pregnancy, smoking during pregnancy, gestational age, birthweight, maternal age, total energy intake, breastfeeding duration, socio-economic status, total maternal polyunsaturated fatty acids intake, history of childhood eye disease, luminosity at eye test sight, childhood lutein and zeaxanthin intake at age four. Ref—referent category; OR—odds ratio.

## Supplementary Materials

The following supporting information can be downloaded at: https://www.mdpi.com/article/10.3390/nu14040872/s1, Figure S1: Directed acyclic graph of hypothesized association between lutein and zeaxanthin intake during pregnancy and visual function in offspring at age 11–12 years; Table S1: Comparison of baseline characteristics of study participants and those lost to follow up or with visual testing issues, based on the Sabadell cohort of the Spanish Childhood and Environment (INMA) project; Table S2: Baseline characteristics of participants according to tertile of energy adjusted lutein and zeaxanthin consumption during the third trimester (32 week assessment) of pregnancy, based on the Sabadell cohort of the Spanish Childhood and Environment (INMA) project; Table S3: Baseline characteristics of participants according to visual acuity and contrast sensitivity status, based on the Sabadell cohort of the Spanish Childhood and Environment (INMA) project; Table S4: Results of Wald test for interaction between lutein and zeaxanthin intake tertiles and selected covariates within multiple regression Model C.
