# Supplementary material for: Lutein and Zeaxanthin Intake during Pregnancy and Visual Function in Offspring at 11–12 Years of Age
Source: Nutrients. 2022 Feb 18;14(4):872. doi: 10.3390/nu14040872 (PMC8876686; doi:10.3390/nu14040872)
Supplement: Supplementary file 1 [file nutrients-14-00872-s001.zip › nutrients-1578317-supplementary.pdf]

Supplementary Materials

Figure S1. Directed acyclic graph of hypothesized association between lutein and zeaxanthin intake during pregnancy and visual function in offspring at age 11-12 years. Graph created using DAGitty software [36].

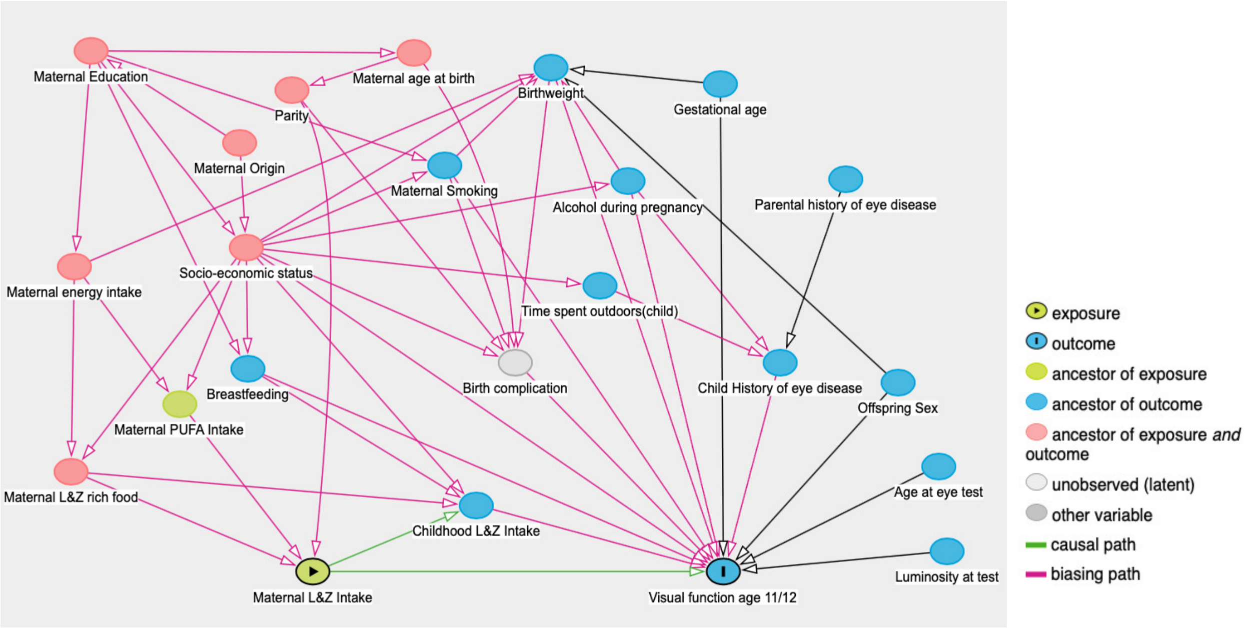

Table S1. Comparison of baseline characteristics of study participants and those lost to follow up or with visual testing issues. Based on the Sabadell cohort of the Spanish Childhood and Environment (INMA) Project.

	n	**Study cohort**	***n***	**Excluded Cohort**	***P* value**
**Maternal Covariates**					
Lutein &Zeaxanthin (energy adjusted) Intake (mg/day), Week 12 Mean (SD)	429	3.5 (2.0)	225	3,6 (2.3)	0.8591[a]
Week 32 Mean (SD)	427	3.4 (2.1)	188	3.5 (2.1)	0.6060[a]
Total energy intake (kcal/day) Week 12 Mean (SD)	429	2050.3 (482.0)	225	2263.0 (600.5)	<0.001[a]
Week 32 Mean (SD)	427	2084.7 (469.5)	188	2305.0 (626.3)	<0.001[a]
Age (years) Mean (SD)	430	31.93 (4.1)	189	30.5 (4.8)	<0.001[a]
Parity, n (%)			224		
Nulliparous	429	250 (58.3)		115 (51.3)	0.09[b]
Parity 1 or more		179 (41.7)		109 (48.7)	
Ethnicity, n (%)			225		
White	430	423 (98.4)		210 (93.3)	0.001[b]
Other Ethnic Group		7 (1.6)		15 (6.7)	
Maternal education, n (%)					
Primary or less	428	102 (23.8)	225	87 (38.7)	<0.001[b]
Secondary		182 (42.5)		97 (43.1)	
Tertiary		144 (33.6)		41 (18.2)	
Social Class, n (%)					
Low	431	183 (42.5)	226	149 (65.9)	<0.001[b]
Medium		144 (33.4)		55 (24.3)	
High		104 (24.1)		22 (9.73)	
Smoking during pregnancy, n (%) No	431	316 (73.3)	226	155 (68.6)	0.201[b]

Yes		115 (26.7)		71 (31.4)	
Alcohol during pregnancy, n (%)					
No	431	333 (77.3)	225	177 (78.7)	0.681[b]
Yes		98 (22.7)		48 (21.3)	
Maternal PUFA Intake (g/day)					
Week 12 Mean (SD)	422	14.2 (3.1)	225	14.1 (3.8)	0.7145[a]
Week 32 Mean (SD)	416	13.5 (2.7)	185	13.4 (3.3)	0.8697[a]
**Child Covariates**					
Sex, n (%)					
Female	431	209 (48.5)	189	98 (51.9)	0.441[b]
Male		222 (51.5)		91 (48.2)	
Gestation (weeks) at birth, Mean (SD)	431	39.7 (1.4)	189	39.5 (2.0)	0.0723[a]
Birthweight Mean (SD)	431	3271.0 (405.3)	186	3159.9 (518.3)	0.0098[a]
Breastfeeding (weeks) Mean (SD)	430	12.8 (9.5)	175	10.9 (9.0)	0.0192[a]
Lutein and Zeaxanthin Intake Age 4 (mg/day) Mean (SD)	377	0.9 (0.5)	63	0.78 (0.4)	0.0107[a]

[a] – p-value from t-Test [b] – p-value from CHI Squared Test [c] – p-value from Fisher Exact Test.

Table S2. Baseline characteristics of participants according to tertile of energy adjusted lutein and zeaxanthin consumption during the third trimester (32 week assessment) of pregnancy. Based on the Sabadell cohort of the Spanish Childhood and Environment (INMA) Project.

	n	Total	1st Tertile (Low)	2nd Tertile (Medium)	3rd Tertile (High)	*P* value
**Maternal Covariates**						
Lutein &Zeaxanthin (energy adjusted) Intake (mg/day), n	427		143	142	142	
Mean (SD)		3.4 (2.1)	1.6 (0.4)	2.9 (0.4)	5.8 (1.9)	
Rank		0.2 -13.1	0.2-2.3	2.3-3.6	3.6-13.1	
Total energy intake (kcal/day) Mean (SD)	427	2084.8 (469.5)	2079.8 (451.6)	2103.9 (458.6)	2070.6 (499.8)	0.827[a]
Age (years) Mean (SD)	429	31.9 (4.1)	30.7 (4.5)	32.9 (3.9)	32.1 (3.7)	<0.0001[a]
Parity, n (%)						
Nulliparous	425	247 (58.1)	93 (65.5)	71 (50.0)	83 (58.9)	0.029[b]
Parity 1 or more		178 (41.9)	49 (34.5)	71 (50.0)	58 (41.1)	
Ethnicity, n (%)						
White	426	419 (98.4)	142 (99.3)	140 (98.6)	137 (97.2)	0.273[c]
Other Ethnic Group		7 (1.6)	1 (0.7)	2 (1.4)	4 (2.8)	
Maternal education, n (%)						
Primary or less	424	100 (23.6)	41 (28.7)	24 (16.9)	35 (25.2)	
Secondary		182 (42.9)	68 (47.6)	61 (43.0)	53 (38.1)	0.016[b]
Tertiary		142 (33.5)	34 (23.8)	57 (40.1)	51 (37.0)	
Social Class, n (%)						
High	427	104 (24.1)	24 (16.8)	43 (30.3)	36 (25.4)	
Medium		143 (33.5)	44 (30.8)	49 (34.5)	50 (35.2)	0.022[b]
Low		181 (42.4)	75 (52.5)	50 (35.2)	56 (39.4)	
Smoking during pregnancy, n (%)						
No	427	313 (73.3)	101 (70.6)	102 (71.8)	110 (77.5	0.380[b]
Yes		114 (26.7)	42 (29.4)	40 (28.2)	32 (22.5)	
Alcohol during pregnancy, n (%)	427					

No		330 (77.3)	106 (74.1)	113 (79.6)	111 (78.2)	0.522[b]
Yes		97 (22.7)	37 (25.9)	29 (20.4)	31 (21.8)	
Maternal PUFA Intake (g/day) Mean (SD)	416	13.5 (2.7)	13.6 (2.6)	13.3 (2.2)	13.5 (3.3)	0.7482[a]
**Child Covariates**						
Sex, n (%)						
Female	427	206 (48.2)	71 (49.7)	70 (49.3)	65 (45.8)	0.770[b]
Male		221 (51.8)	72 (50.4)	72 (50.7)	77 (54.2)	
Prematurity (<37 weeks gestation)						
No	427	417 (97.7)	139 (97.2)	140 (98.6)	138 (97.2)	0.782[c]
Yes		10 (2.3)	4 (2.8)	2 (1.4)	4 (2.8)	
Gestation (weeks) at birth, Mean (SD)	427	39.8 (1.4)	39.8 (1.3)	39.7 (1.5)	39.6 (1.3)	0.8097[a]
Birthweight Mean (SD)	427	3273.2 (403.9)	3283.7 (416.2)	3275.0 (399.4)	3260.7 (398.4)	0.8894[a]
Predominant Breastfeeding (weeks) Mean (SD)	426	12.8 (9.5)	12.6 (9.4)	13.6 (9.3)	12.2 (9.7)	0.4192[a]
Lutein and Zeaxanthin Intake Age 4 (mg/day) Mean (SD)	373	0.9 (0.5)	0.8 (0.4)	0.9 (0.5)	1.1 (0.5)	0.0001[a]
**Vision Covariates**						
Parental History of Eye Disease[d] n (%)						
None	418	84 (20.1)	28 (20.0)	31 (22.3)	25 (18.0)	0.263[b]
One Parent		179 (42.8)	63 (45.0)	49 (35.3)	67 (48.2)	
Both parents		155 (37.1)	49 (35.0)	59 (42.5)	47 (33.8)	
Childhood history of eye disease[d] n (%)						
No	419	329 (78.5)	117 (83.6)	106 (75.7)	106 (76.3)	0.203[b]
Yes		90 (21.5)	23 (16.4)	34 (24.3)	33 (23.7)	
Age at Eye Test (years) Mean (SD)	427	11.2 (0.5)	11.1 (0.5)	11.1 (0.5)	11.3 (0.5)	0.0023[a]

[a] – p-value from ANOVA [b] – p-value from CHI Squared Test [c] – p-value from Fisher Exact Test [d] – Includes any reported ametropia but excluding presbyopia.

Table S3. Baseline characteristics of participants according to visual acuity and contrast sensitivity status. Based on the Sabadell cohort of the Spanish Childhood and Environment (INMA) Project.

	n	Visual Acuity (Below 20th Centile)	Visual Acuity (Above 20th Centile)	P value	*n*	Contrast Sensitivity (Below 20th Centile)	Contrast Sensitivity (Above 20th Centile)	*P* value
**Maternal Covariates**		*n* = 89	*n* = 341			*n* = 93	*n* = 335	
Lutein & Zeaxanthin (energy adjusted) Intake (mg/day)								
Week 12, Mean (SD)	428	3.7 (2.0)	3.5 (2.0)	0.3694[a]	426	3.6 (1.9)	3.5 (2.0)	0.8605[a]
Week 32, Mean (SD)	426	3.5 (2.2)	3.4 (2.0)	0.6877[a]	424	3.8 (1.9)	3.4 (2.5)	0.1040[a]
Total energy intake (kcal/day)								
Week 12 Mean (SD)	428	2039.5 (400.1)	2054.2 (502.1)	0.7984[a]	426	2089.1 (450.7)	2040.8 (491.9)	0.3966[a]
Week 32 Mean (SD)	426	2106.6 (434.7)	2079.2 (479.4)	0.6253[a]	424	2099.4 (455.6))	2078.7 (474.2)	0.7067[a]
Age (years) Mean (SD)	429	32.2 (4.2)	31.8 (4.1)	0.4165[a]	427	32.5 (3.9))	31.8 (4.2)	0.1128[a]
Parity, n (%)								
Nulliparous	428	45 (50.6)	204 (60.2)	0.102[b]	426	46 (50.0)	202 (60.5)	0.071[b]

Parity 1 or more		44 (49.4)	135 (39.8)			46 (50.0)	132 (39.5)	
Ethnicity, n (%)								
White	429	89 (100.0)	33 (97.9)	0.353[c]	427	91 (97.9)	329 (98.5)	0.649[c]
Other Ethnic Group		0 (0.0)	7 (2.1)			2 (2.2)	5 (1.5)	
Maternal education, n (%)								
Primary or less	427	28 (31.5)	74 (21.9)		425	30 (32.3)	71 (21.4)	
Secondary		35 (39.3)	146 (43.2)	0.163[b]		38 (40.9)	142 (42.8)	0.067[b]
Tertiary		26 (29.2)	118 (34.9)			25 (26.9)	119 (35.8)	
Social Class, n (%)								
High	430	16 (18.0)	88 (25.8)		428	19 (20.4)	85 (25.4)	
Medium		31 (34.8)	112 (32.8)	0.295[b]		29 (31.2)	115 (34.3)	0.354[b]
Low		42 (47.2)	141 (41.4)			45 (48.4)	135 (40.3)	
Smoking during pregnancy, n (%)								
No	430	66 (74.2)	250 (73.3)	0.872[b]	428	63 (67.7)	250 (74.6)	0.185[b]
Yes		23 (25.8)	91 (26.7)			30 (32.3)	85 (25.4)	
Alcohol during pregnancy, n (%)								
No	430	73 (82.0)	259 (76.0)	0.224[b]	428	73 (78.5)	257 (76.7)	0.718[b]
Yes		16 (18.0)	82 (24.1)			20 (21.5)	78 (23.3)	
Maternal PUFA Intake (g/day)								
Week 12 Mean (SD)	419	14.7 (4.1)	14.1 (2.8)	0.0921[a]	419	14.4 (3.1)	14.1 (3.1)	0.4757[a]
Week 32 Mean (SD)	415	13.6 (2.5)	13.4 (2.8)	0.5628[a]	413	13.6 (4.5)	13.4 (2.5)	0.5067[a]
**Child Covariates**								
Prematurity (<37 weeks gestation) n (%)								
No	430	87 (97.8)	333 (97.7)	1.000[c]		90 (96.8)	328 (97.9)	0.458[c]
Yes		2 (2.3)	8 (2.4)			3 (3.2)	7 (2.1)	
Gestation (weeks) at birth, Mean (SD)	430	39.6 (1.5)	39.8 (1.3)	0.4199[a]	428	39.6 (1.5)	39.8 (1.3)	0.3318[a]
Birthweight Mean (SD)	430	3225.4 (441.4)	3282.6 (395.7)	0.2368[a]	428	3228.0 (425.1)	3283.32 (400.9)	0.2459[a]
Birthweight, n (%)								
<3000,		26 (29.2)	86 (25.22)			31 (33.3)	81 (24.2)	
3000-3500		44 (49.4)	157 (46.0)	0.364[b]		38 (40.9)	161 (48.1)	0.197[b]
>3500		19 (21.4)	98 (28.7)			24 (25.8)	93 (27.8)	
Predominant Breastfeeding (weeks)								
Mean (SD)	429	12.3 (9.5)	13.0 (9.5)	0.5695[a]	427	13.5 (9.8)	12.7 (9.4)	0.4699[a]
n (%)								
None		20 (22.5)	67 (17.7)			19 (20.4)	67 (20.1)	
0-16 weeks		27 (30.3)	106 (31.2)	0.828[b]		27 (29.3)	106 (31.7)	0.942[b]
16-24 weeks		34 (38.2)	126 (37.1)			35 (37.6)	124 (37.1)	
>24 weeks		8 (9.0)	41 (12.1)			12 (12.9)	37 (11.1)	
Fruit and Vegetable Intake Age 4 (g/day) Mean (SD)	376	223.4 (114.2)	209.2 (103.6)	0.2918[a]	374	200.2 (108.2)	215.0 (102.7)	0.2509[a]
Lutein and Zeaxanthin Intake Age 4 (mg/day) Mean (SD)	376	0.94 (0.47)	0.91 (0.46)	0.6718[a]	374	0.90 (0.44)	0.92 (0.55)	0.7750[a]
**Vision Covariates**								
Parental History of Eye Disease[d] n (%)								
None	421	14 (15.9)	71 (21.3)		419	16 (17.4)	68 (20.8)	
One Parent		28 (31.8)	152 (45.7)	0.004[b]		37 (40.2)	143 (43.7)	0.461[b]
Both parents		46 (52.3)	110 (33.0)			39 (42.4)	116 (35.5)	

Childhood history of eye disease[d] n (%)<br>No<br>Yes	422	34 (38.6)<br>54 (61.4)	297 (88.9)<br>37 (11.1)	P<0.0001[b]	420	70 (75.3)<br>23 (24.7)	261 (79.8)<br>66 (20.18)	0.344[b]
Age at Eye Test (years)<br>Mean (SD)	430	11.1 (0.5)	11.2 (0.5)	0.3569[a]	428	11.1 (0.5)	11.2 (0.5)	0.3479[a]

[a] – p-value from t-test between groups [b] – p-value from CHI Squared Test [c] – p-value from Fisher Exact Test [d] – Includes any reported ametropia but excluding presbyopia.

Table S4. Results of Wald test for interaction between lutein and zeaxanthin intake tertiles and selected covariates within multiple regression Model C.

	**Contrast Sensitivity Model C**	**Visual Acuity Model C**
**Week 12**	p-value for interaction	p-value for interaction
Social class	0.1571	0.4506
Breastfeeding	0.8683	0.0648
Parity	0.2759	0.3267
Sex	0.2260	0.6241
**Week 32**		
Social class	0.8121	0.4059
Breastfeeding	0.5159	0.8075
Parity	0.0052	0.0254
Sex	0.7227	0.4381
